# Liver Fibrosis Stages Affect Organic Cation Transporter 1/2 Activities in Hepatitis C Virus-Infected Patients

**DOI:** 10.3390/ph17070865

**Published:** 2024-07-01

**Authors:** Matheus De Lucca Thomaz, Carolina Pinto Vieira, Juciene Aparecida Caris, Maria Paula Marques, Adriana Rocha, Tiago Antunes Paz, Rosamar Eulira Fontes Rezende, Vera Lucia Lanchote

**Affiliations:** 1 Department of Clinical Analysis, Food Science and Toxicology, School of Pharmaceutical Sciences of Ribeirão Preto, University of São Paulo, Ribeirão Preto 14040-903, Brazil; matheusdethomaz@usp.br (M.D.L.T.); carolina.vieira@unifenas.br (C.P.V.); juciene@outlook.com (J.A.C.); mpcosta@fcfrp.usp.br (M.P.M.); drirocha@fcfrp.usp.br (A.R.); tiagoapaz@gmail.com (T.A.P.); 2 Division of Gastroenterology, Department of Internal Medicine, School of Medicine of Ribeirão Preto, University of São Paulo, Ribeirão Preto 14049-900, Brazil; rosamarrezende@uol.com.br; 3 Reference Center, Hepatitis Outpatient Clinic, Municipal Health Secretary, Ribeirão Preto 14049-900, Brazil

**Keywords:** chronic hepatitis C, metformin, organic cation transporter, inflammation, pharmacokinetics, liver fibrosis, clinical pharmacology, membrane transporters

## Abstract

This study aims to evaluate the impact of liver fibrosis stages of chronic infection with hepatitis C virus (HCV) on the in vivo activity of organic cation transporters (hepatic OCT1 and renal OCT2) using metformin (MET) as a probe drug. Participants allocated in Group 1 (*n* = 15, mild to moderate liver fibrosis) or 2 (*n* = 13, advanced liver fibrosis and cirrhosis) received a single MET 50 mg oral dose before direct-acting antiviral (DAA) drug treatment (Phase 1) and 30 days after achieving sustained virologic response (Phase 2). OCT1/2 activity (MET AUC_0–24_) was found to be reduced by 25% when comparing the two groups in Phase 2 (ratio 0.75 (0.61–0.93), *p* < 0.05) but not in Phase 1 (ratio 0.81 (0.66–0.98), *p* > 0.05). When Phases 1 and 2 were compared, no changes were detected in both Groups 1 (ratio 1.10 (0.97–1.24), *p* > 0.05) and 2 (ratio 1.03 (0.94–1.12), *p* > 0.05). So, this study shows a reduction of approximately 25% in the in vivo activity of OCT1/2 in participants with advanced liver fibrosis and cirrhosis after achieving sustained virologic response and highlights that OCT1/2 in vivo activity depends on the liver fibrosis stage of chronic HCV infection.

## 1. Introduction

It is estimated that 58 million people worldwide have chronic infection with hepatitis C virus (HCV), with approximately 1.5 million new infections each year, which, in severe cases, can progress to liver cirrhosis and/or hepatocellular carcinoma. Chronic HCV infection treatment is carried out through the administration of direct-acting antiviral (DAA) drugs, with a cure rate of over 95% [1].

Chronic HCV infection creates a microenvironment with high levels of proinflammatory cytokines, including tumor necrosis factor-alpha (TNF-α), interleukin-6 (IL-6), interferon-gamma (IFN-γ), interleukin-1α (IL-1α), interleukin-8 (IL-8), and interleukin-2 (IL-2), among others [2,3,4,5]. Liver fibrosis during chronic HCV infection, which is assessed by the METAVIR score [6], is associated with fibrogenesis, including collagen deposition and abnormal remodeling of the extracellular matrix [3,7].

Drug transporters are membrane-bound proteins that play an important role in drug absorption, distribution, and excretion. There is ample and clinical evidence that infection and inflammation associated with the induction of proinflammatory cytokines modulate drug-metabolizing enzyme activities, while convincing in vivo evidence for the modulation of drug transporters, including organic cation transporters (hepatic OCT1 and renal OCT2), has not been demonstrated [8,9,10,11]. Induction or inhibition of these drug transporters can alter the pharmacokinetics of several drugs, such as antihypertensive agents (pindolol), histamine antagonists (cimetidine), anticancer agents (platinum agents), smoking cessation aids (varenicline), and antidiabetic agents (metformin) [12]. Currently, regulatory agencies, such as the Food and Drug Administration (United States), the European Medicines Agency, and the International Transporter Consortium, already recommend the assessment of the impact of developing drugs on OCT1/2 activities [13,14,15,16].

Nevertheless, information regarding the impact of HCV infection on OCT1/OCT2 activities remains predominantly limited to in vitro investigations by transcriptomics [17,18,19] and quantitative proteomics [20,21,22,23]. These studies showed a reduction in mRNA levels of the OCT1 gene (*SLC22A1*) and a decrease in its protein expression in HCV infection when compared to control groups [17,18,19,21,22,23].

Considering that the downregulation of OCT1 may reduce the hepatic uptake of drugs and that the correlation between mRNA levels and protein expression is not always satisfactory, in the present study, the in vivo activity assessment of OCT1/OCT2 was conducted through phenotyping using metformin (MET) as a probe [24,25,26,27,28,29] in a single subtherapeutic oral dose of 50 mg [24].

MET has been characterized in vitro as a substrate of the drug transporters OCT1/2, and its metabolism in humans is negligible, allowing for investigation of transporter activity without the potential for enzyme inhibition to confound the results. MET uptake into the liver and kidneys depends on OCT1 and OCT2 activities, respectively. So, MET plasma exposure (area under the plasma concentration–time curve; AUC_0–24_) has been used to characterize OCT1/2 in vivo activity following single oral doses [24,25,26,27,28,29].

In this study, participants with chronic HCV infection, genotypes 1 and 3, were investigated in two phases. Phase 1 occurred one day prior to the beginning of treatment with DAA drugs, while Phase 2 took place within 30 days after confirming sustained virologic response (HCV-RNA undetectable 12 weeks post-treatment completion). It is noteworthy that Phase 2 was conducted following the complete elimination of DAA drugs used in the treatment of chronic HCV infection

## 2. Results

Table 1 presents the anthropometric, biochemical, and hematological parameters of the participants (*n* = 28) diagnosed with chronic HCV infection.

The participants of the present study (*n* = 28) were classified according to the stage of liver fibrosis (METAVIR score [6]) as F0 + F1 (*n* = 7), F2 (*n* = 8), F3 (*n* = 4), and F4 (*n* = 9). The participants were distributed into Group 1 (F0 + F1 + F2) and Group 2 (F3 + F4); see Table 1. Most participants showed high levels of AST and ALT before DAA drug treatment (Phase 1), confirming the liver damage caused by chronic HCV infection [30], where ALT levels in Group 2 were significantly higher than in Group 1 (Mann–Whiney test for unpaired data, *p*-value < 0.05).

Regarding Phase 2 (within 30 days after confirming sustained virologic response) of the study, it was observed that, for both AST and ALT, only two participants had plasma levels outside the reference values. In addition, significant reductions in the levels of these biomarkers were observed in Phase 2 when compared to Phase 1 (Wilcoxon test for paired data, *p*-values < 0.05), Table 1.

Considering that MET is distributed to adipose tissue [31], it is worth highlighting that some participants were overweight or obese (BMI ≥ 25 kg/m^2^): seven participants in Group 1 and nine in Group 2, with median (range) values of 24 (20–45, Group 1) and 30 kg/m^2^ (21–39, Group 2), respectively. However, Groups 1 and 2 were not considered statistically different (Mann–Whitney test for unpaired data, *p*-value > 0.05).

**Figure 1:**
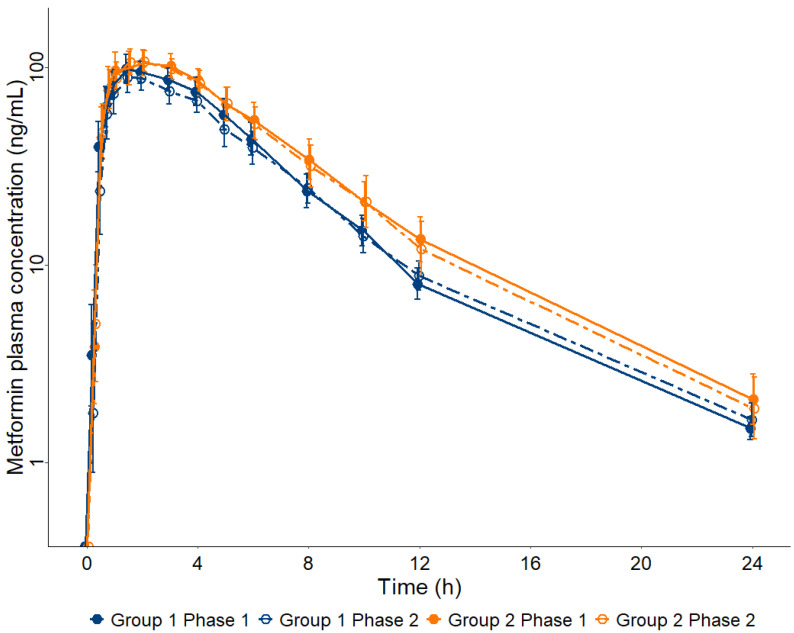
Metformin (MET) plasma concentration–time curves up to 24 h after a single oral dose of 50 mg in participants diagnosed with chronic HCV infection prior to treatment with direct-acting antivirals (Phase 1) and after obtaining a sustained virologic response (Phase 2), allocated into two groups according to their METAVIR score, Group 1 (F0 + F1 + F2, *n* = 15) and Group 2 (F3 + F4, *n* = 13). Data are presented as geometric mean and 90% confidence interval.

Figure 1 presents the geometric mean and CI 90% for MET plasma concentration–time curves for the participants of the study distributed in Groups 1 and 2 along Phases 1 and 2. The method of metformin analysis in plasma by LC-MS/MS (Supplementary Materials) showed a Lower Limit of Quantification (LLOQ) of 0.25 ng/mL, allowing the analysis of all plasma samples (*n* = 840) collected from 28 patients in Phases 1 and 2 of the study until 24 h after a single oral dose of 50 mg MET.

Table 2 shows the distribution of MET pharmacokinetic parameters using the Shapiro–Wilk test. Data were classified as log-normal distribution, with the exception of the t_max_ values classified as non-parametric distribution.

Table 3 presents the geometric means (95% CI) or medians (IQR P5-P95) of the MET pharmacokinetics parameters for Groups 1 and 2 in both phases (1 and 2) of the study.

Before treatment with DAA drugs (Phase 1), MET pharmacokinetic parameters do not differ between Groups 1 (*n* = 15; F0 + F1 + F2) and 2 (*n* = 13; F3 + F4) (Student’s *t*-test for unpaired samples, *p*-value > 0.05), Table 3.

However, after the sustained virologic response (Phase 2), AUC_0–24_ and AUC_0–∞_ are increased in Group 2 when compared to Group 1, whereas Vd/F and Cl/F are decreased in Group 2 when compared to Group 1 (Student’s *t*-test for unpaired samples, *p*-value < 0.05), Table 3.

In addition, MET pharmacokinetic parameters do not show significant differences between Phases 1 and 2 of the study (Student’s *t*-test for paired samples, *p*-value > 0.05), neither for Group 1 nor for Group 2; Table 3.

**Figure 2:**
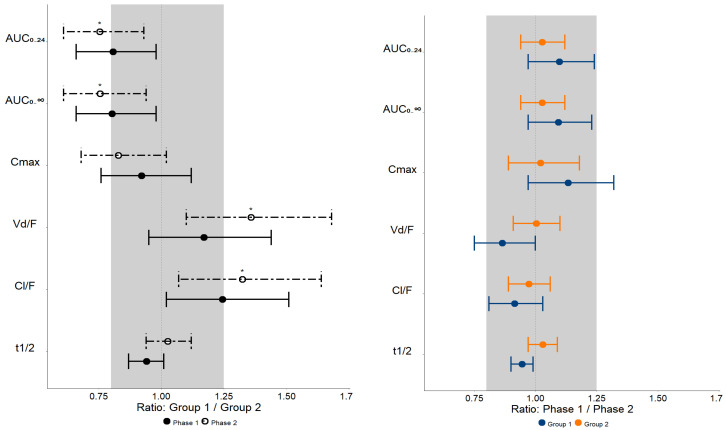
Bioequivalence plots of metformin pharmacokinetics parameters observed in patients with mild to moderate liver fibrosis (Group 1, F0 + F1 + F2, *n* = 15) and severe liver fibrosis and cirrhosis (Group 2, F3 + F4, *n* = 13) prior to treatment with direct-acting antivirals drugs (Phase 1) and after obtaining a sustained virologic response (Phase 2). The shaded area represents the bioequivalence range (0.8–1.25). AUC—area under the plasma concentration–time curve; C_max_—maximum plasma concentration; Vd/F—apparent distribution volume; Cl/F—apparent clearance; t_1/2_—elimination half-life. Data are presented as the geometric mean ratio (CI 90%) * *p* < 0.05.

Table 4 and Figure 2 show the geometric mean ratios Group 1/Group 2 and Phase 1/Phase 2 (90% CI) and their respective *p*-values when the MET pharmacokinetics parameters are plotted along the bioequivalence range (0.8–1.25).

Regarding Groups 1 and 2 comparisons in both phases of the study, the 90% CI values of the geometric mean ratios (Group 1/Group 2) of MET AUC_0–24_, AUC_0–∞,_ C_max_, Vd/F, and Cl/F are not contained within the bioequivalence range of 0.8–1.25, implying that there is no clinical equivalence between Groups 1 and 2 (Table 4 and Figure 2) in Phases 1 and 2.

However, regarding Phases 1 and 2 comparisons in both groups of the study, the 90% CI values of the geometric mean ratios (Phase 1/Phase 2) of MET pharmacokinetic parameters (AUC_0–24_, AUC_0–∞_, and Cl/F) are within the bioequivalence range of 0.8–1.25, implying that there is clinical equivalence between Phases 1 and 2 (Table 4 and Figure 2) in Groups 1 and 2.

**Figure 3:**
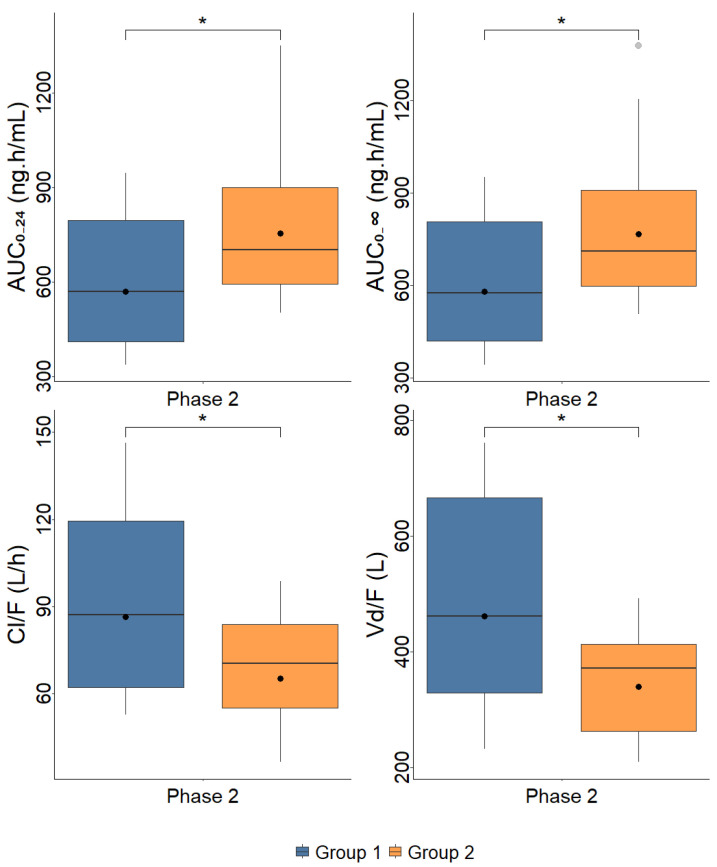
Boxplots of metformin pharmacokinetics parameters observed in patients with mild to moderate liver fibrosis (Group 1, F0 + F1 + F2, *n* = 15) and severe liver fibrosis and cirrhosis (Group 2, F3 + F4, *n* = 13) prior to treatment with direct-acting antivirals drugs (Phase 1) and after obtaining a sustained virologic response (Phase 2). AUC—area under the plasma concentration–time curve; C_max_—maximum plasma concentration; Vd/F—apparent distribution volume; Cl/F—apparent clearance; t_max_—time to reach C_max_; t_1/2_—elimination half-life. Dots represent geometric means * *p* < 0.05.

Figure 3 presents the boxplots of MET pharmacokinetics parameters, comparing the participants from each group in the different phases of the study.

## 3. Discussion

In this study, participants diagnosed with chronic HCV infection (*n* = 28) were investigated one day prior to the beginning of treatment with DAA drugs (Phase 1) and within 30 days after confirming sustained virologic response (Phase 2). Since this study was conducted with Brazilian participants, who are highly heterogeneous and admixed, it is important to emphasize the influence of demographic covariates, such as age and body surface area, but also the proportion of African ancestry, which combined with the pharmacogenetic marker OCT1-R61C related to a low expression of the OCT1 trans-porter, explain 29.7% of the variability in MET plasma exposure (AUC_0–48_) [32].

MET, being a high-solubility and low-permeability drug (Biopharmaceutical Classification System: BCS class III), has its distribution to both liver (OCT1) and kidneys (OCT2) determined by transporter activity [33,34]. As MET was administered orally, the observed pharmacokinetic parameters reflect not only the activity of hepatic OCT1 and renal OCT2 transporters but also those located on the apical, primarily OCT3 and PMAT, and on the basolateral, OCT1, membrane of enterocytes, as well as MATE1K and MATE2K on the apical membrane of renal tubular cells [34].

MET undergoes minimal hepatic metabolism and insignificant biliary excretion, so its total clearance does not depend on hepatic clearance, allowing for investigation of transporter inhibition without the potential for enzyme inhibition or altered hepatic blood flow to confound the results [34,35,36]. Although the hepatic artery blood flow is increased in hepatitis C, the portal vein blood flow remains unchanged; however, it tends to decrease with the progression of liver fibrosis and the presence of cirrhosis, with portosystemic shunting being one of the reasons [37,38,39].

MET is mainly cleared (approximately 80%) by renal elimination mediated by OCT2 (expressed on the basolateral membrane of renal tubule cells) into tubule cells and by MATE1 and MATE2 (expressed on the apical membrane of renal tubule cells) into urine [25,34]. MET pharmacokinetics exhibits circadian rhythm dependent on glomerular filtration rate, renal blood flow, and OCT2 transporter activity, with higher values of total apparent clearance (Cl/F) in the morning compared to the evening [40,41]. So, in the present study, all participants received a single oral dose of 50 mg MET in the morning as a probe of OCT1/2 in both phases of the study (Phases 1 and 2).

Considering that MET bioavailability is dose-dependent [34], the AUC_0–24_ values obtained in the present study with a single oral dose of 50 mg are comparable only to that reported by Nguyen et al. (2020) [24] at the same dose of 50 mg. Before treatment with DAA drugs (Phase 1), plasma exposure values of MET AUC_0–24_ [624.82 ng·h/mL (531.09–735.09) and 774.57 ng·h/mL (641.69–934.81), respectively, for Groups 1 and 2] (Table 3) are close to the MET AUC_0–24_ of 606 ng·h/mL previously reported following a 50 mg MET dose in healthy participants [24].

Before treatment with DAA drugs (Phase 1), MET plasma exposure did not differ between Groups 1 and 2 (Table 3), suggesting that the activity of OCT1/2 transporters does not change between different stages of liver fibrosis. However, the 90% CI of the geometric mean ratios (Group 1/Group 2) of MET AUC_0–24_ of 0.66–0.98 is not contained within the bioequivalence range of 0.8–1.25, implying that there is no clinical equivalence between the Groups 1 and 2 before DAA treatment (Table 4) probably because of the small number of investigated patients for this analysis.

Considering that Phase 2 of the current study was conducted after achieving sustained virologic response, approximately 12 weeks after the completion of treatment with DAA drugs (sofosbuvir + daclatasvir ± ribavirin, or ombitasvir + ritonavir + dasabuvir ± ribavirin, or sofosbuvir + simeprevir ± ribavirin) and considering that the elimination half-lives of DAA drugs and their metabolites vary between 0.4 h and 12 days in patients diagnosed with chronic HCV infection [42], the possibility of pharmacokinetic interaction of these drugs with MET can be excluded.

After sustained virologic response (Phase 2), plasma exposure values of MET (AUC_0–24_) are increased in patients with advanced liver fibrosis/cirrhosis (Group 2; *n* = 13) when compared to patients with mild to moderate liver fibrosis (Group 1; *n* = 15); (Table 3). On the other hand, other MET pharmacokinetic parameters, such as Vd/F and Cl/F, are decreased in Group 2 when compared to Group 1. In addition, the geometric mean ratios (90% CI) (Group 1/Group 2) in Phase 2 for AUC_0–24_ 0.75 (0.61–0.93), AUC_0–∞_ 0.76 (0.61–0.94), Vd/F 1.36 (1.10–1.68), and Cl/F 1.32 (1.07–1.64) are not contained within the clinical equivalence range of 0.8–1.25 (Table 4 and Figure 2), implying increased MET plasma exposure due to its limited hepatic (MET distribution dependent on OCT1) and/or renal distribution (MET distribution dependent on OCT2) in participants of Group 2 when compared to Group 1, indicating that patients with severe stages of liver fibrosis and cirrhosis (Group 2 = F3 + F4) have an approximately 25% reduction in in vivo activity of OCT1/2 drug transporter (AUC_0–24_ ratio 0.75 [0.61–0.93]) after sustained virologic response.

It is important to highlight that this result agrees with previous in vitro data. Ogasawara et al. (2010) [17] showed that the mRNA levels of OCT1 were 35% decreased in patients of the F4 stage (cirrhosis) compared to non-cirrhosis patients (F0, no fibrosis; F1 portal fibrosis without septa; F2, portal fibrosis with rare septa; and F3, numerous septa without cirrhosis). When all patients with liver fibrosis were compared to the control group, a 30% reduction in mRNA levels of OCT1 was observed. Nakai et al. (2008) [19] also reported that the OCT1 mRNA levels in liver biopsy samples of patients of the F3 stage were decreased when compared to the patients of the F1 stage. In addition, these authors showed that the OCT1 mRNA levels in HepG2 cells were significantly decreased according to TNF-α treatment. Studies using quantitative proteomics also showed decreased protein expression (per gram of liver) of OCT1 in liver tissues from patients with hepatitis C cirrhosis classified as Child-Pugh classes A, B, and, especially, C when compared to the control group [21,22,23].

Although the present study does not present proinflammatory cytokines plasma concentrations for the investigated participants, the reduction in the in vivo activity of OCT1/2 observed in patients with severe stages of liver fibrosis and cirrhosis after achieving sustained virologic response (Phase 2) may be related to the inflammatory response in chronic HCV infection, in addition to the fibrotic process, which can also alter OCT1/2 transporter activity [18,43,44,45].

However, considering that MET plasma exposure (AUC_0–24_) does not show significant differences between Phases 1 and 2 of the study (Table 3), neither for Group 1 nor for Group 2, it is possible to infer that DAA treatment does not alter the activity of OCT1/2 transporters. In addition, the values of the 90% CI of the geometric mean ratios (Phase 1/Phase 2) for Groups 1 and 2, respectively—for AUC_0–24_, 1.10 (0.97–1.24) vs. 1.03 (0.94–1.12); for AUC_0–∞_, 1.09 (0.97–1.23) vs. 1.03 (0.94–1.12); and for C_max,_ 1.13 (0.97–1.32) vs. 1.02 (0.89–1.18)—are contained within the bioequivalence range of 0.8–1.25 (Table 4). These results may be explained by the absence of regression in the liver fibrosis stage, as demonstrated by Radmanic et al. (2022) [46], where it was observed that only 30% of participants showed improvement in liver fibrosis status when examining the relationship between sustained virologic response (12 weeks after DAA drug treatment) and the fibrosis stage. In addition, the cited authors did not find significant differences in serum levels of TNF-α and IL-6 before DAA drug treatment and after sustained virologic response. Montaldo et al. (2021) [47], investigating circulating extracellular vesicles from patients infected with HCV before treatment with DAA drugs and six months after obtaining sustained virologic response, highlighted that long-term fibrosis may progress even with HCV clearance mediated by DAA drug treatment, which may explain the equivalence of the parameters evaluated in the two phases of this study.

The main limitation of this study is the absence of pharmacogenetic data; it is important to emphasize the influence of single nucleotide polymorphisms (SNPs) on OCT1 activity, where reduced MET uptake in hepatocytes has been demonstrated in participants carrying the OCT1-M420del (rs72552763) allele, with a frequency of 20% in white Americans and 5% in African Americans, and OCT1-R61C (rs12208357), with a frequency of 7.2% in white individuals, showing lower expression of the drug transporters, suggesting a contribution of these polymorphisms to reduced therapeutic response to MET, whereas regarding the frequency of this SNP in the Brazilian population, it is described as 7% [32,48].

## 4. Methods and Materials

### 4.1. Clinical Study

The study was approved by the Research Ethics Committees of the School of Pharmaceutical Sciences of Ribeirão Preto—University of São Paulo (CAAE: 60161116.4.0000.5403) and by the university hospital of the Ribeirão Preto Medical School—University of São Paulo (CAEE: 60161116.4.3001.5440).

The participants were previously informed about the purpose of the study, its duration, the procedures involved, and the potential risks, and then they signed the Free and Informed Consent Form. The participants were free to refuse to participate or withdraw their consent at any stage of the research without penalty or prejudice to their care and/or treatment.

The study included participants diagnosed with chronic HCV infection, genotypes 1 and 3, who were recruited by convenience sampling at the Reference Center, Hepatitis Outpatient Clinic, Municipal Health Secretary, Ribeirão Preto, São Paulo, Brazil, from August 2017 to January 2020. Both male and female participants, aged 18 years and older, classified according to the degree of liver fibrosis/cirrhosis (F0 + F1, F2, F3, and F4), and who signed the Free and Informed Consent Form were included. Patients with any of the following clinical conditions were excluded from the investigation: HIV or hepatitis B coinfection; the presence of other comorbidities such as chronic kidney disease (stages 1 to 5), diabetes mellitus, or hypothyroidism, among others; and the current use of medications that may inhibit or induce membrane transporters OCT1/2.

Anthropometric, biochemical, and hematological parameters were routinely assessed by the university hospital via electronic medical records.

The participants of the study (*n* = 28) were evaluated by transient hepatic elastography (FibroScan^®^; Echosens, Paris, France) and/or liver biopsy distributed into two groups according to the stage of liver fibrosis. The groups were classified according to the METAVIR score [6]: Group 1—patients with early liver fibrosis or its absence (F0 + F1 = 7) and moderate liver fibrosis (F2 = 8), *n* = 15; Group 2—patients with severe liver fibrosis (F3 = 4) and cirrhosis (F4, Child Pugh-A, *n* = 9), *n* = 13. The participants were treated with different DAA drugs combinations: sofosbuvir + daclatasvir ± ribavirin, ombitasvir + ritonavir + dasabuvir ± ribavirin, or sofosbuvir + simeprevir ± ribavirin.

The investigation comprised two phases: Phase 1, one day before the beginning of DAA drugs treatment; Phase 2, up to 30 days after the evaluation of sustained virologic response, conducted 12 weeks after the end of treatment, when HCV-RNA should be undetectable.

**Figure 4:**
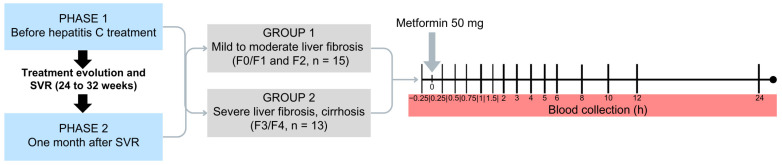
Design of the clinical study including patients with chronic HCV infection classified as mild to moderate liver fibrosis (Group 1, F0/F1, F2, *n* = 15) or severe liver fibrosis and cirrhosis (Group 2, F3, F4, *n* = 13) prior to treatment with direct-acting antivirals drugs (Phase 1) and after obtaining a sustained virological response (SVR, Phase 2).

In Phase 1 of the study, participants were admitted to the Clinical Research Unit of the local hospital in a fasting state for 8 h; they received a single oral dose of 50 mg of MET hydrochloride (gelatin capsule) with 250 mL of water. The participants remained fasting for 4 h after drug administration. Serial blood samples were collected in heparinized tubes at 0 (15 min before MET administration), 15, 30, and 45 min and 1, 1.5, 2, 3, 4, 5, 6, 8, 10, 12, and 24 h after drug administration. The blood samples were centrifuged at 1800× *g* for 10 min (Figure 4). Plasma aliquots were separated and stored at −80 °C until analysis. In Phase 2 of the study, participants underwent the same procedure described in Phase 1.

### 4.2. Metformin Analysis in Plasma

MET was analyzed in plasma samples by LC-MS/MS using a previously developed (Tables S1 and S2 and Figures S1 and S2 in Supplementary Materials) and validated method (Table S3) with linearity in the range of 0.25–250 ng/mL, as described in detail in the Supplementary Materials [15,49,50].

### 4.3. Power Analysis

The sample size (*n*) was determined using the software PS: Power and Sample Size Calculation version 3.1.6 using data from the area under the plasma concentration–time curve (AUC_0–24_ ± standard deviation of 650 ± 163 ng·h/mL) of MET obtained in the investigation of 15 healthy participants who received a single oral dose of MET (50 mg) [24]. Our hypothesis was that the plasma exposure of MET obtained before the DAA drug treatment would be approximately 30% higher than those obtained 1 month after obtaining the sustained virologic response. To determine the sample size, the significance level was set at 5%, and the power of the test was set at 80%, resulting in the inclusion of at least 12 participants who completed both phases.

### 4.4. Pharmacokinetics Analysis

Pharmacokinetic parameters were calculated using Phoenix WinNonlin™ software, version 8.3.3.33 (Certara USA, Inc., Princeton, NJ, USA). The area under the plasma concentration–time curve (AUC_0–24_) of MET was evaluated using non-compartmental analysis with the linear trapezoidal interpolation method with extrapolation to infinity (AUC_0–∞_) using the formula C_last_/K_el_, where C_last_ represents the last observed plasma concentration and K_el_ is the elimination rate constant.

### 4.5. Statistical Analysis

The distributions of the pharmacokinetic parameters, such as AUC_0–24_, AUC_0–∞_, maximum plasma concentration (C_max_), apparent volume of distribution (Vd/F), apparent clearance (Cl/F), time to reach C_max_ (t_max_), and elimination half-life (t_1/2_), were evaluated using the Shapiro–Wilk statistical normality test. According to the distribution data, the pharmacokinetic parameters were classified as normal, log-normal, or non-parametric.

Statistically significant differences between Phases 1 and 2 (paired Student’s *t*-test or Wilcoxon test) and Groups 1 and 2 (unpaired Student’s *t*-test or Mann–Whitney test) were defined when the *p*-values were below the threshold of 0.05. The geometric means ratios of AUC and C_max_ between phases or between groups were considered clinical equivalent if their 90% confidence intervals (90% CI) were completely contained within the range of 0.8–1.25 [51].

Statistical analysis and determination of the geometric mean, coefficient of variation, and confidence interval of the experimental data were performed using the R software, version 4.3.2, and the figures were plotted with the ggplot2 package.

## 5. Conclusions

This study shows a reduction of approximately 25% in the in vivo activity of OCT1/2 in participants with severe stages of liver fibrosis and cirrhosis after achieving sustained virologic response and highlights that OCT1/2 in vivo activity depends on the liver fibrosis stage. Therefore, dose adjustment for OCT1/2 substrates with low therapeutic index should observe the stage of liver fibrosis and cirrhosis in patients with chronic HCV infection.

## Figures and Tables

**Table 1:** Anthropometric and biochemical parameters of the participants diagnosed with chronic HCV infection allocated, according to their METAVIR score, to Group 1 (F0 + F1 + F2, *n* = 15) and Group 2 (F3 + F4, *n* = 13), prior to treatment with direct-acting antivirals drugs (Phase 1) and after obtaining a sustained virologic response (SVR, Phase 2). Data are presented as median and range.

	Reference Values	Phase of the Study	Group 1 (*n* = 15)		Group 2 (*n* = 13)	
			Median	Range	Median	Range
Age (years)	-	-	48	35–65	56	37–68
Sex	-	-	Male = 9, female = 6		Male = 7, female = 6	
Weight (kg)	-	-	64	55–119	79	53–113
Height (m)	-	-	1.64	1.50–1.78	1.68	1.58–1.82
BMI (kg/m^2^)	-	-	24	20–45	30	21–39
METAVIR score	-	-	F0 + F1 (*n* = 7), F2 (*n* = 8)		F3 (*n* = 4), F4 (*n* = 9)	
Glycemia (mg/dL)	70–99	1	87	53–96	84	78–118
		2	88	79–102	91	81–140
Total proteins (g/dL)	6.0–8.0	1	7.5	6.0–8.7	8.0	6.8–8.8
		2	7.6	6.6–8.0	7.7	7.3–8.7
Albumin (g/dL)	3.5–5.5	1	4.2	3.0–4.6	3.9	3.1–4.9
		2	4.0	3.6–4.5	4.3	3.3–4.8
Serum creatinine (mg/dL)	0.6–1.2	1	0.8	0.7–1.1	0.9	0.5–1.6
		2	0.8	0.6–1.1	0.9	0.6–1.6
GFR (mL/min)	≥70	1	87	53–174	104	42–215
		2	94	65–232	124	45–179
ALT (U/L)	10–40	1	48 *^#^	20–237	80 *^#^	31–845
		2	27 ^#^	14–63	27 ^#^	9–49
AST (U/L)	15–40	1	37 ^#^	20–85	47 ^#^	24–392
		2	21 *^#^	11–42	25 *^#^	20–60
Total bilirubin (mg/dL)	0.1–1.0	1	0.6	0.3–65.4	0.6	0.3–3.0
		2	0.5	0.2–1.0	0.7	0.4–1.4
Direct bilirubin (mg/dL)	<0.3	1	0.2	0.1–0.4	0.2	0.1–0.9
		2	0.1	0.1–0.2	0.2	0.1–0.4
INR	0.8–1.0	1	0.93	0.76–1.02	0.98	0.91–1.3
		2	0.95	0.91–0.99	0.98	0.93–1.25
Urea (mg/dL)	16–40	1	28	20–35	32	18–78
		2	27	25–47	28	20–77
Direct-acting antiviral drugs	-	-	Daclatasvir, dasabuvir, ombitasvir, ribavirin, ritonavir, sofosbuvir, veruprevir		Daclatasvir, dasabuvir, ombitasvir, ribavirina, ritonavir, simeprevir, sofosbuvir, veruprevir	

* Mann–Whitney test for unpaired data (Group 1 versus Group 2), *p* < 0.05. ^#^ Wilcoxon test for paired data (Phase 1 vs. Phase 2), *p* < 0.05.

**Table 2:** Distribution of metformin pharmacokinetic parameters using the Shapiro–Wilk test.

		Data		Log-Transformed Data	
	PHASE	*p*-Value	W Value	*p*-Value	W Value
AUC_0–∞_	1	0.4028	0.9627	0.9951	0.9906
	2	0.1057	0.9392	0.8989	0.9822
AUC_0–24_	1	0.5075	0.9672	0.9946	0.9904
	2	0.1251	0.9421	0.9153	0.9830
C_max_	1	0.1356	0.9435	0.1712	0.9475
	2	0.1727	0.9476	0.7731	0.9770
Vd/F	1	0.01216	0.9011	0.1819	0.9485
	2	0.01794	0.9083	0.3853	0.9618
Cl/F	1	0.1723	0.9476	0.9951	0.9906
	2	0.2135	0.9513	0.8989	0.9822
t_max_	1	0.0011	0.8540	0.0217 *	0.9117
	2	0.0100	0.8975	0.0493 *	0.9261
t_1/2_	1	0.0497	0.9263	0.1743	0.9478
	2	0.0163	0.9065	0.1729	0.9476

* p < 0.05, Shapiro–Wilk test.

**Table 3:** Pharmacokinetic parameters of metformin following a single oral dose of 50 mg in participants diagnosed with chronic HCV infection allocated into two groups according to their METAVIR score: Group 1 (F0 + F1 + F2, *n* = 15) and Group 2 (F3 + F4, *n* = 13). Groups 1 and 2 are compared in each phase of treatment: before pharmacological treatment with direct-acting antiviral drugs (Phase 1) and after (Phase 2) obtaining a sustained virologic response. Data are presented as geometric mean (95% CI).

	Phase	Group 1			Group 2		
		*n*	Geometric Mean (95% CI)	CV (%)	*n*	Geometric Mean (95% CI)	CV (%)
AUC_0–∞_ (ng·h/mL)	**1**	15	632.76 (538.45–743.59)	29.77	13	787.38 (651.32–951.86)	32.18
	**2**	15	578.87 * (478.69–700.02)	35.35	13	766.34 * (631.97–929.28)	32.73
AUC_0–24_ (ng·h/mL)	**1**	15	624.82 (531.09–735.09)	29.99	13	774.57 (641.79–934.81)	31.89
	**2**	15	569.02 * (470.01–688.89)	35.57	13	754.06 * (623.89–911.39)	32.15
C_max_ (ng/mL)	**1**	15	109.53 (93.95–127.69)	28.24	13	118.93 (98.05–144.25)	32.77
	**2**	15	96.63 (78.95–118.26)	37.73	13	116.54 (99.39–136.64)	26.80
Vd/F (L)	**1**	15	398.26 (330.47–479.96)	34.67	13	340.12 (283.29–408.35)	30.96
	**2**	15	460.87 * (372.4–570.36)	39.96	13	339.16 * (289.65–397.14)	26.57
Cl/F (L/h)	**1**	15	79.02 (67.24–92.86)	29.77	13	63.50 (52.53–76.77)	32.18
	**2**	15	86.38 * (71.43–104.45)	35.35	13	65.25 * (53.81–79.12)	32.73
t_max_ (h) ^#^	**1**	15	1.50 (1.00–3.00)	55.70	13	1.50 (1.00–3.00)	49.14
	**2**	15	2.00 ^#^ (1.00–3.00)	42.83	13	2.00 ^#^ (1.50–3.00)	43.78
t_1/2_ (h)	**1**	15	3.49 (3.35–3.65)	7.76	13	3.71 (3.42–4.03)	13.70
	**2**	15	3.70 (3.48–3.94)	11.28	13	3.60 (3.27–3.97)	15.94

* *p*-value < 0.05 Student *t*-test for unpaired samples, Group 1 vs. Group 2; AUC: area under the plasma concentration–time curve; C_max_: maximum plasma concentration; Vd/F—apparent volume of distribution; Cl/F—apparent clearance; t_max_—time to reach C_max_; t_1/2_—elimination half-life. ^#^ t_max_ is presented as median (IQR P5-P95); CV—coefficient of variation.

**Table 4:** Pharmacokinetic parameters of metformin following a single oral dose of 50 mg in participants diagnosed with chronic HCV infection allocated into two groups according to their METAVIR score: Group 1 (F0 + F1 + F2, *n* = 15) and Group 2 (F3 + F4, *n* = 13). Groups 1 and 2 are compared in each phase of treatment, before pharmacological treatment with direct-acting antiviral drugs (Phase 1) and after (Phase 2) obtaining a sustained virologic response. Data are presented as the geometric mean ratio (CI 90%).

	Phase 1/Phase 2		Group 1/Group 2	
	Group	Ratio	Phase	Ratio
AUC_0–∞_	**1**	1.09 (0.97–1.23)	**1**	0.80 (0.66–0.98)
	**2**	1.03 (0.94–1.12)	**2**	0.76 (0.61–0.94)
AUC_0–24_	**1**	1.10 (0.97–1.24)	**1**	0.81 (0.66–0.98)
	**2**	1.03 (0.94–1.12)	**2**	0.75 (0.61–0.93)
C_max_	**1**	1.13 (0.97–1.32)	**1**	0.92 (0.76–1.12)
	**2**	1.02 (0.89–1.18)	**2**	0.83 (0.68–1.02)
Vd/F	**1**	0.86 (0.75–1.00)	**1**	1.17 (0.95–1.44)
	**2**	1.00 (0.91–1.10)	**2**	1.36 (1.10–1.68)
Cl/F	**1**	0.91 (0.81–1.03)	**1**	1.24 (1.02–1.51)
	**2**	0.97 (0.89–1.06)	**2**	1.32 (1.07–1.64)
t_max_	**1**	0.75 (0.45–2.20)	**1**	1.00 (---)
	**2**	0.75 (0.45–2.00)	**2**	1.00 (---)
t_1/2_	**1**	0.94 (0.90–0.99)	**1**	0.94 (0.87–1.01)
	**2**	1.03 (0.97–1.09)	**2**	1.03 (0.94–1.12)

## Data Availability

Data is contained in the paper.

## Supplementary Materials

The following supporting information can be downloaded at: https://www.mdpi.com/article/10.3390/ph17070865/s1, Table S1: Transitions, cone, and collision energies of metformin (MET) and internal standard metformin-d6 (MET-d6); Table S2: Plasma concentrations of metformin (MET) in quality control (QC) samples; Table S3: Validation parameters of the metformin analysis in plasma; Figure S1: Mass spectra of metformin (A) and internal standard metformin-d6 (B); Figure S2: Chromatograms of metformin (A) and internal standard metformin-d6 (B) in blank plasma, metformin (C) and internal standard metformin-d6 (D) in blank plasma enriched at the LLOQ concentration, and metformin (E) and internal standard metformin-d6 (F) in plasma from a patient with hepatitis C collected 1.5 h after the administration of a single oral dose of 50 mg of metformin enriched with internal standard metformin-d6.
