# Supplementary material for: Liver Fibrosis Stages Affect Organic Cation Transporter 1/2 Activities in Hepatitis C Virus-Infected Patients
Source: Pharmaceuticals (Basel). 2024 Jul 1;17(7):865. doi: 10.3390/ph17070865 (PMC11280093; doi:10.3390/ph17070865)
Supplement: Supplementary file 1 [file pharmaceuticals-17-00865-s001.zip › pharmaceuticals-3076065-supplementary.pdf]

Supplementary Material

Method of analysis of metformin in plasma by UPLC-MS/MS

The method of analysis of metformin in plasma was developed and validated according to previous data, using metformin-d6 as internal standard, and ultra-performance liquid chromatography coupled with tandem mass spectrometry (UPLC-MS/MS) [1,2].

1. Materials and Methods

1.1 Solvents and reagents

MET hydrochloride (99.8%) was obtained from the British Pharmacopoeia (London, UK), while metformin hydrochloride-d6 (95%) was acquired from Supelco (Darmstadt, Germany). Methanol (J.T. Baker, Xalostoc, Mexico) and acetonitrile (Biograde, Anápolis, GO, Brazil) were purchased as HPLC grade, while ammonium acetate (Sigma-Aldrich, Cotia, SP, Brazil) and glacial acetic acid (Merck, Darmstadt, Germany) were acquired as analytical grade. Water used throughout the analysis was prepared from the Milli-Q Plus purification system obtained from Millipore Corp. (Bedford, MA, USA).

1.2 Standard solutions

Stock solutions of MET and internal standard (IS) metformin-d6 (MET-d6) were prepared in methanol at the concentrations of 1 mg/mL and 0.2 mg/mL, respectively. Calibration standards were prepared by diluting the MET stock solution in methanol to concentrations of 0.50, 1.00, 2.00, 5.00, 10.0, 25.0, 50.0, 100, 250, and 500 ng/mL. The IS working solution (10 ng/mL) was prepared in methanol from serial dilutions of the MET-d6 stock solution. Quality control (QC) samples were prepared in blank plasma at 4 different concentrations: lower limit of quantification (LLOQ): 0.25 ng/mL; low-quality control (LQC): 0.5 ng/mL; medium-quality control (MQC): 125 ng/mL; and high-quality control (HQC): 200 ng/mL. The dilution quality control (DQC) was prepared at a concentration of 500 ng/mL and diluted 1:10 with blank plasma. Standard solutions of MET and the QCs were stored at -20 °C until use.

1.3 Sample preparation

Aliquots of 50 µL of blank plasma were transferred to microtubes and added with 25 µL of the IS solution (MET-d6, 10 ng/mL in methanol) and 150 µL of methanol. The tubes were vortexed for 1 minute and then centrifuged at 4°C for 15 minutes at 21,500 g. Aliquots of 100 µL of the supernatants were transferred to inserts, and 10 µL were injected into the chromatographic system.

1.4 Chromatographic Analysis

The determination of MET in plasma was performed using ultra-performance liquid chromatography coupled with tandem mass spectrometry (UPLC-MS/MS), consisting of an Acquity UPLC® H-Class quaternary pump, an Acquity UPLC® Sample Manager - FTN autosampler equipped with an Acquity Sample Organizer, and an XEVO TQ-S® triple quadrupole mass spectrometer with an ESI Zspray™ interface, all from Waters Corp. (Milford, MA, USA). The chromatographic analysis was carried out on an Acquity UPLC HSS T3 column (1.8 µm, 2.1 x 100 mm, Waters, Dublin, Ireland), with the same pre-column. The mobile phase was composed by aqueous ammonium acetate buffer solution (pH 4.8, 2 mM) and acetonitrile (97:3, v/v), at a flow rate of 0.25 mL/min. The MS/MS analyses were performed in positive ionization mode. The capillary voltage in the ESI was set at 3.00 kV. The source temperature was set at 150°C, while the desolvation temperature was maintained at 450°C with a nitrogen flow rate of 1000 L/h. Argon gas was used as the collision gas at a flow rate of 0.19 mL/min. The cone voltage was maintained at 25 V with a nitrogen flow rate of 150 L/h. The analyses were performed in multiple reaction monitoring mode. The protonated ions [M + H]+ and their respective product ions were monitored in the transitions of 130 → 60 m/z for MET and 136 → 60 m/z for MET-d6, with a collision energy of 11 eV in both cases (Table S1). Data acquisition and sample quantification were performed using MassLynx® software version 4.1 (Micromass, Manchester, UK).

2 Method Validation

The analytical method was validated for selectivity, linearity, matrix effect, precision, accuracy, and short-term, post-processing, and freeze and thaw

stabilities, according to the Guideline on bioanalytical method validation of the European Medicines Agency [3].

2.1 Calibration Curve

The calibration curve for MET in plasma was prepared by enriching 50 µL aliquots of blank plasma with 25 µL of each MET standard solution in methanol to obtain plasma concentrations of 0.25, 0.50, 1.00, 2.5, 5.00, 12.5, 25.0, 50.0, 125, and 250 ng/mL. Calibration curves were considered acceptable when at least 75% of the calibration standards showed deviations less than or equal to 15% from the nominal values, except for the LLOQ concentrations, where deviations up to 20% from the nominal values were accepted.

2.2 Matrix Effect

The matrix effect was evaluated by analyzing eight samples of blank plasma obtained from different participants, including four normal, two lipemic, and two hemolyzed samples. Aliquots of blank plasma were processed and then enriched with IS and MET at LQC and HQC concentrations (Table S2). Standard solutions in methanol at LQC and HQC concentrations, along with the IS solution, were also analyzed. The matrix factor normalized by IS (MFNIS) was calculated using the equation MFNIS = (peak area of the analyte in plasma/peak area of the internal standard in plasma)/(peak area of the analyte in solution/peak area of the internal standard in solution). The absence of matrix effect was considered when the coefficient of variation of all MFNIS values were less than 15%.

2.3 Precision and Accuracy

Within-run and between-run precision and accuracy were determined by analyzing blank plasma samples enriched with five different MET concentrations corresponding to LLOQ, LQC, MQC, HQC, and DQC values (Table S2).

Within-run precision and accuracy were evaluated using six replicates analyzed within a single analytical run, while between-run precision and accuracy were evaluated using six replicates analyzed in three separate runs. Accuracy and precision were expressed as the relative error (RE%) and coefficient of variation (CV%), respectively. The acceptable limit for precision and accuracy is ±15% of

the nominal concentration, except for the LLOQ sample, which should be within ±20%.

2.4 Selectivity

The selectivity of the method was evaluated by analyzing six aliquots of blank plasma obtained from different participants, including four normal samples, one lipemic sample, and one hemolyzed sample. The method is considered selective when no interfering peaks greater than 20% for MET and 5% for the IS, respectively, are detected at their respective retention times compared to the peaks observed in the processed LLOQ sample (Table S2).

2.5 Carryover effect

The carryover effect was assessed based on the analysis of three injections of the same blank plasma sample, one before and two immediately after injecting a processed sample at the upper limit of quantification (ULQ, 250 ng/mL). The responses of interfering peaks at the retention time of the analyte and IS should be less than 20% and 5%, respectively, compared to the processed LLOQ samples (0.25 ng/mL).

3 Results

The method was developed using the XEVO TQ-S® triple quadrupole mass spectrometer. The protonated ions [M + H]+ and their respective product ions were monitored in the transitions 130 → 60 for MET and 136 → 60 for the internal standard MET-d6 (Figure S1). MET and the internal standard were eluted from the Acquity UPLC HSS T3 column with a mobile phase composed of an aqueous solution of 2 mM ammonium acetate buffer (pH 4.8) and acetonitrile (97:3, v/v) with a retention time of 2.08 min (Figure S2). The method demonstrated linearity in the plasma concentrations range from 0.25 to 250 ng/mL. The concentrations of the calibration standards in plasma were determined using linear regression analysis with weighting ($1/x^2$) in the equation $y = ax + b$. The matrix effect of the method was within the established limits, with no significant interferences in the ionization of MET and the internal standard MET-d6. The results of within-run and between-run precision and accuracy were considered satisfactory, as well as the stability results, which were within the acceptance criteria of the analytical method

(Table S3). Regarding the selectivity of the method, no significant interferences from any endogenous compounds were identified in the blank plasma samples at the retention times of MET and the internal standard MET-d6 (Figure S2). No residual effect was observed.

Table S1 – Transitions, cone, and collision energies of metformin (MET) and internal standard metformin-d6 (MET-d6).

Analyte	Transition (m/z)	Cone energy (V)	Collision energy (eV)
**MET**	130 > 60	25	11
**MET-d6**	136 > 60	25	11

Table S2 - Plasma concentrations of metformin (MET) in quality control (QC) samples.

	Concentration (ng/mL)				
**MET in plasma**	**LLOQ**	**LQC**	**MQC**	**HQC**	**DQC**
	0.25	0.5	125	200	500

LLOQ: lower limit of quantification; LQC: low-quality control; MQC: medium-quality control; HQC: high-quality control; DQC: dilution quality control (1:10).

Table S3 - Validation parameters of the metformin analysis in plasma.

**MFNIS (CV%)**	LQC:9.83<br>HQC:1.50<br>LQC and HQC:7.24
**Linearity (ng/mL)**	0.25 - 250
**Linear equation**	y = 0.18899 * x + 0.0547899
**r^2**	0.991719

	**Precision (CV%)**	**Accuracy (RE%)**
	Within-run (n = 6)	
**LLOQ**	6.62	-1.33
**LQC**	8.39	7.33
**MQC**	4.57	1.53
**HQC**	3.38	-7.07
**DQC**	3.62	-1.08
	Between-run (n = 3)	
**LLOQ**	8.80	-2.44
**LQC**	10.55	-1.11
**MQC**	4.65	-0.05
**HQC**	2.72	-7.09
**DQC**	4.05	-4.18
	Freeze-thaw stability	
**LQC**	6.26	5.50
**HQC**	1.53	-11.59
	Short-term stability	
**LQC**	3.02	8.00
**HQC**	7.02	-4.00
	Post-processing stability	
**LQC**	11.68	1.60
**HQC**	2.09	-6.49

MFNIS: matrix factor normalized by internal standard; CV: coefficient of variation, expressed as a percentage; RE: relative error, expressed as a percentage; LLOQ: lower limit of quantification (0.25 ng/mL); LQC: low-quality control (0.5 ng/mL); MQC: medium-quality control (125 ng/mL); HQC: high-quality control (200 ng/mL); DQC: dilution quality control (500 ng/mL, 1:10 dilution).

Figure S1 - Mass spectra of metformin (A) and internal standard metformin-d6 (B).

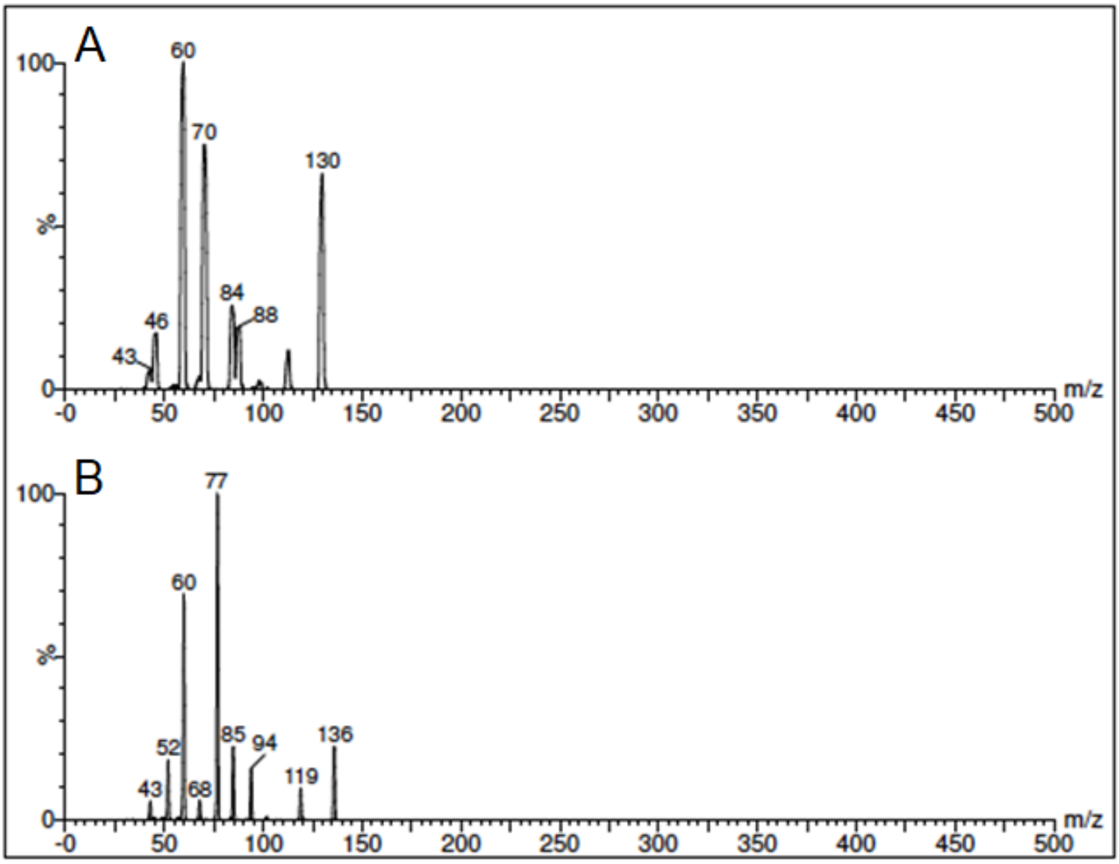

Figure S2 - Chromatograms of metformin (A) and internal standard metformin-d6 (B) in blank plasma, metformin (C) and internal standard metformin-d6 (D) in blank plasma enriched at the LLOQ concentration, and metformin (E) and internal standard metformin-d6 (F) in plasma from a patient with hepatitis C collected 1.5 h after the administration of a single oral dose of 50 mg of metformin enriched with internal standard metformin-d6.

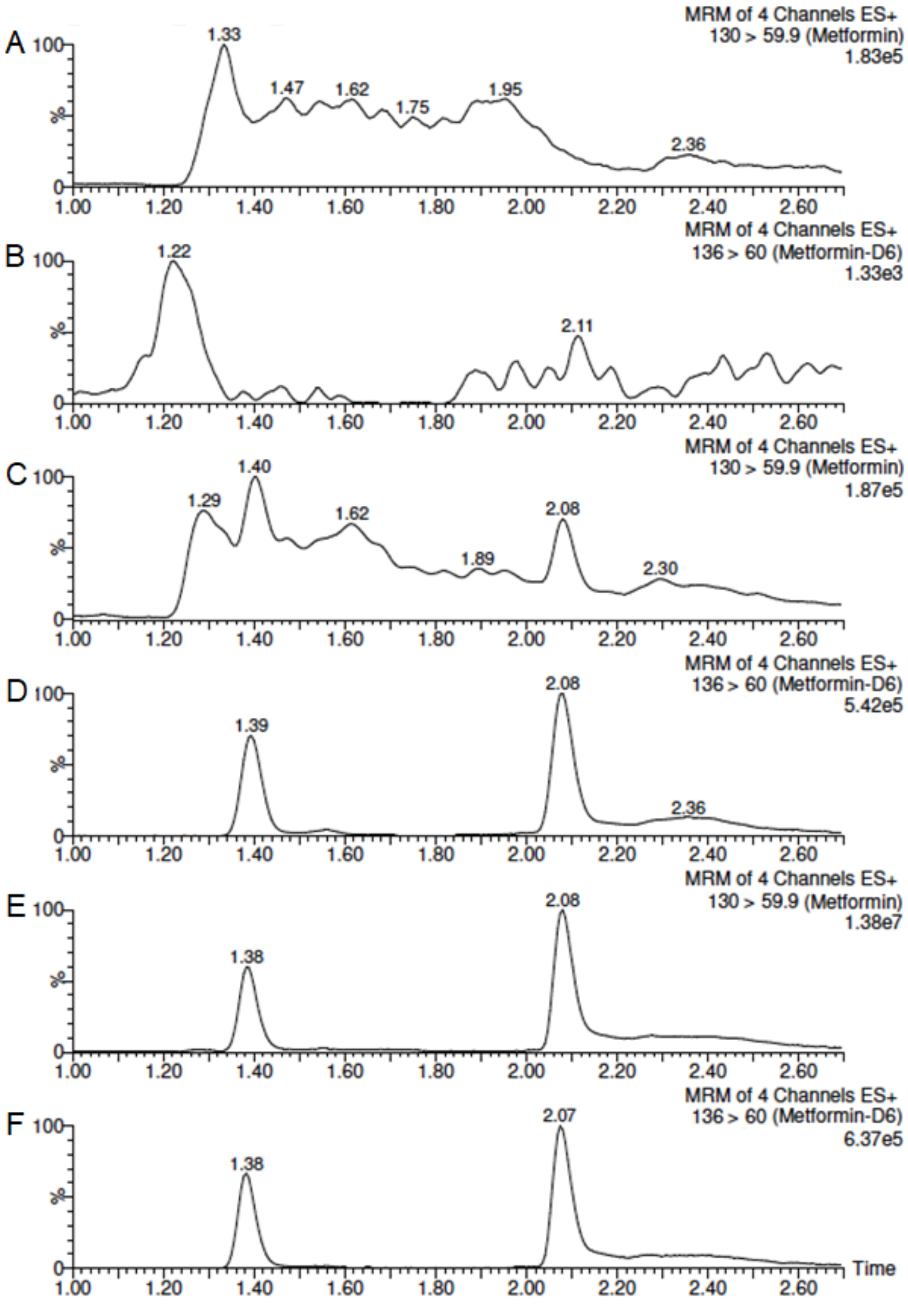

References

1-Kumar PP, Murth TEGK, Basaveswara Rao MV. Development, validation of liquid chromatography-tandem mass spectrometry method for simultaneous determination of rosuvastatin and metformin in human plasma and its application to a pharmacokinetic study. J Adv Pharm Technol Res. 2015; 6(3):118-24. doi: 10.4103/2231-4040.157982.

2-Chaudhari K, Wang J, Xu Y, Winters A, Wang L, Dong X, Cheng EY, Liu R, Yang S-H. Determination of metformin bio-distribution by LC-MS/MS in mice treated with a clinically relevant paradigm. PLoS One. 2020; 15, e0234571. https://doi.org/10.1371/journal.pone.0234571

3-European Medicines Agency Committee for Medicinal Products for Human Use, 2022. ICH Guideline M12 on drug interaction studies Step 2b Available online: <https://www.ema.europa.eu/en/documents/scientific-guideline/draft-ich-guideline-m12-drug-interaction-studies-step-2b_en.pdf>
